# Precipitation of Inorganic Salts in Mitochondrial Matrix

**DOI:** 10.3390/membranes10050081

**Published:** 2020-04-27

**Authors:** Jerzy J. Jasielec, Robert Filipek, Krzysztof Dołowy, Andrzej Lewenstam

**Affiliations:** 1 Faculty of Materials Science and Ceramics, AGH University of Science and Technology, Al. Mickiewicza 30, 30-059 Krakow, Poland; rof@agh.edu.pl; 2 Department of Physics and Biophysics, Warsaw University of Life Sciences—SGGW, Nowoursynowska 159, 02-776 Warszawa, Poland; krzysztof_dolowy@sggw.edu.pl; 3 Johan Gadolin Process Chemistry Centre, Centre of Process Analytical Chemistry and Sensor Technology (ProSens), Åbo Akademi University, Biskopsgatan 8, 20500 Åbo-Turku, Finland; alewenst@abo.fi

**Keywords:** mitochondrion, calcium carbonates, calcium phosphates, calcium polyphosphates, ATP production, hypoxia, ischemia, pre-conditioning

## Abstract

In the mitochondrial matrix, there are insoluble, osmotically inactive complexes that maintain a constant pH and calcium concentration. In the present paper, we examine the properties of insoluble calcium and magnesium salts, such as phosphates, carbonates and polyphosphates, which might play this role. We find that non-stoichiometric, magnesium-rich carbonated apatite, with very low crystallinity, precipitates in the matrix under physiological conditions. Precipitated salt acts as pH buffer, and, hence, can contribute in maintaining ATP production in ischemic conditions, which delays irreversible damage to heart and brain cells after stroke.

## 1. Introduction

Mitochondria are responsible for the adenosine triphosphate (ATP) production in eukaryotic organisms. They are also involved in Ca^2+^ signalling, lipid metabolism, heat production, reactive oxygen species production and apoptosis [1]. It is becoming increasingly apparent that mitochondria can accumulate, store and release a large amount of calcium ions under a variety of physiological and pathological conditions such as epilepsy [2], ischemia [3,4] and concussive brain injury [5]. 

**Scheme 1:**
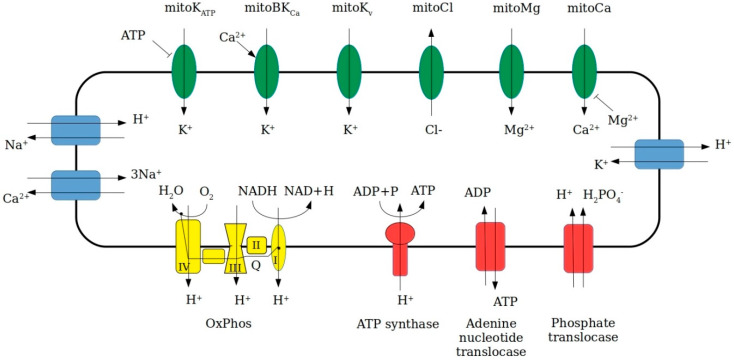
Ion channels, exchangers, pumps and oxidative phosphorylation chain of the inner mitochondrial membrane: green—uniporters, blue—exchangers, yellow—four complexes of oxidative phosphorylation chain, and red—ATP synthasome. There are more than one type of mitoBK_Ca_, mitoKv or mitoCl channels.

The mitochondrial matrix is surrounded by two membranes. The outer membrane acts as a molecular sieve, while the inner membrane is ion-selective. The major function of the mitochondrial inner membrane is the formation of the electrochemical proton gradient, known as proton motive force [6], utilizing the energy produced by burning fats and sugars. The proton motive force is then used by ATP synthase to synthetize ATP from adenosine diphosphate (ADP) and inorganic phosphate. In the mitochondrial inner membrane there are numerous ion channels and ion exchangers which are involved in the transport of calcium, magnesium, potassium, sodium, protons, phosphates and other ions. The most relevant transporters in the mitochondrial inner membrane are depicted in Scheme 1. Due to the very high electric potential difference (inside negative), the mitochondrial matrix tends to accumulate calcium ions, which form a complex of calcium phosphates and carbonates. Of interest in this report are free calcium and magnesium ions, and the composition of the solid aggregates precipitating in the matrix.

The free concentration of Ca^2+^ and Mg^2+^ depends on complex chemistry which is not easy to decode because of thermodynamic and kinetic factors. The capacity of isolated mitochondria to accumulate calcium up to total matrix concentration of 0.5 M to 0.8 M, has been established more than half a century ago [7,8,9,10,11]. However, the free calcium concentration in the range of 0.17 to 5 μM has been reported by numerous studies using fluorescent Ca^2+^ indicators (see Table 1).

In mitochondria calcium has important physiological role in stimulation of ATP synthesis via activation of several Ca^2+^-sensitive enzymes (pyruvate-, α-ketoglutarate-, isocitrate dehydrogenases), ATP synthase and adenine nucleotide translocase [12,13]. Matrix enzymes are activated by calcium of concentration in the micromolar range, i.e., [Ca^2+^]_matrix_ = 0.5 to 2 μM [14]. Calcium can be accumulated in or released from the mitochondrion, depending on the proton motive force of the mitochondrion and cytoplasmic free calcium concentration. 

In the presence of phosphates, calcium appears to form an insoluble and osmotically inactive complex, which buffer calcium concentration [7]. Amorphous granules containing calcium and phosphorus were identified within the mitochondria of osteoclasts [15], chondrocytes [16,17], osteoblasts [18], osteocytes [19,20,21], calcifying cartilage [22], and mineralizing bone [23,24,25]. It is impossible to investigate the nature of the calcium phosphates in the matrix directly, due to their instant dissociation when mitochondria are disrupted and due to their ability to transform during fixation or drying for chemical analysis [26]. Additionally, it is reasonable to presume that these complexes in the matrix are inert, to some extend at least. Even more importantly, the composition of mitochondrial granules is still largely unknown [27] and it is based on several hypotheses. Thomas and Greenawalt [28] suggested that inorganic granules found in mitochondria are a colloidal, sub-crystalline precursor of calcium-deficient hydroxyapatite. Lehninger [8] and later Nicholls and Chalmers [29] assumed the formation of amorphous tricalcium phosphate, while Kristian et al. [27] suggested the formation of brushite and non-stoichiometric amorphous calcium phosphate with Ca/P ratio equal 1.47. Dołowy, in the most recent hypothesis [30], assumed that brushite turns into isoclasite via all intermediate forms of calcium phosphate minerals forming a polymer-like structure. 

The inorganic cations present in the mitochondrial matrix include Na^+^, K^+^, Ca^2+^ and Mg^2+^. All sodium and potassium salts are soluble. Calcium and magnesium, on the other hand, can form a variety of insoluble salts. The solubility products of calcium and magnesium hydroxides are relatively high (K_Ca(OH)2_ = 6.5 × 10^−6^ and K_Mg(OH)2_ = 5.6 × 10^−12^), therefore in considered system Ca(OH)_2_ and Mg(OH)_2_ will dissociate completely. In this work, the chemical equilibria of numerous compounds, including calcium and magnesium carbonates, phosphates and polyphosphates are intentionally examined. Based on the solubility products and other properties of these salts, we identify the reason for the pH buffering effect, i.e., the calcium hydroxide buffer effect in the mitochondrial matrix.

## 2. Calculations and Data

### 2.1. Mitochondrial pH, Ionic Concentrations and Activities

One must realize the meaning of pH in the systems with very low volumes. The amount of protons can be calculated as *n*_H+_ = *N_A_* × *V* × 10^−pH^, where *N_A_* = 6.02 × 10^−23^ is the Avogadro number and *V* represents volume. The mitochondrion is an organelle with a diameter of c.a. 1 μm, hence the volume of ~1 fL. The mitochondrial matrix occupies 90% of mitochondrial volume. With pH_mat_ = 8.2, one obtains 3.4 protons in the matrix. This indicates that water is too weakly dissociated to be a direct donor or acceptor of protons at very low biological volumes (even if one can still define the pH formally) and that protons can be shuttled to reaction centers by other biomolecules, whose concentrations are not constrained by the solubility product of water [31]. These protons may be provided by carbonic or phosphoric acid or dissolution of their salts.

The molecular probes used for pH measurements, like e.g., SNARF-1 (seminaphtharhodafluor) [32,33,34] or mutants of green fluorescent protein [35,36,37], report their own protonation state and not the concentration of free protons in the solution.

The values of pH and the concentrations of inorganic ions, measured in mitochondria using various methods, are listed in Table 1. The mitochondrion is a complex organelle in which pH and concentrations of ions fluctuate in response to metabolic processes and perturbations of the cytosolic environment [38]. Mitochondrial pH is reported to be in the range of 7.2 to 8.4 with an average value of 7.8 (see Table 1). However, when mitochondria are loaded with succinate, the pH can reach values close to 9 [39]. The concentration of calcium in mitochondria usually varies between 0.17 to 5 µM (see Table 1). However, in the case of mitochondria of firing axon, it can reach values as high as 24 µM [40]. Furthermore, the concentrations of ions are interdependent, e.g., calcium effluxes from mitochondria can depend on the concentration of sodium [41].

The driving force for the transition of dissolved matter from a supersaturated solution to the equilibrium solid phase, is given by the change of standard free energy Δ*G*^0^ = −*RT*ln*S* , where *S* = *Q*/*K_sp_* is supersaturation defined as the ratio of the ion activity product (*Q*) of constituent ions in solution over its equilibrium solubility product (*K_sp_*). If *S* > 1, the solution is supersaturated, and precipitation may occur. If *S* < 1, the solution is undersaturated, and there will be no precipitation.

The activity product of a salt *A_x_B_y_* is defined as:

(1) $$Q={\left\{A\right\}}^{x}\times {\left\{B\right\}}^{y}=\gamma_{A}{}^{x}\times \gamma_{B}{}^{y}\times {\left[A\right]}^{x}\times {\left[B\right]}^{y}$$

where {*A*} and {*B*} are the activities, [*A*] and [*B*] are the concentrations, *γ_A_* and *γ_B_* are the activity coefficients of ions *A* and *B*, respectively.

In ideal solution, the activity coefficients of all ions equal one. However, in the concentrated solutions *γ_i_* can significantly vary from unity and strongly depends on the ionic strength of the solution defined as:

(2) $$I=0.5\sum z_{i}{}^{2}c_{i}$$

where *c_i_* is the concentration of *i*-th ion and *z_i_* represents its charge. It is impossible to precisely calculate the ionic strength of the mitochondrial matrix. Taking into account just the inorganic ions present in the matrix (see Table 1) and assuming similar concentration of inorganic ions, one can assume *I* of the matrix to be in the range of 0.4 to 1 M. The extended Debye-Hückel can predict the activity coefficient in a solution with an ionic strength up to 100 mM and Davies equation is valid up to an ionic strength of 500 mM [42]. In this work, we use the modified Davies equation, which can correctly predict the values of the activity coefficient in solutions with ionic strength up to 1 M [42]:

**Table 1:** Mitochondrial concentrations of selected inorganic ions.

	Value	Measuring Technique	Type of Cell	Ref.
pH	7.14	SNARF	pig heart	[32]
	7.8	BCECF	beef heart	[43]
	7.65–8.1	SNARF	beef heart	[33]
	8.0–8.9	DMO distribution	rat heart	[39]
	7.40–7.51	DMO distribution	beef heart	[44]
	7.74–7.95	ratiometric pericam	motor nerve of *Drosophila* fly larvae	[40]
	7.7	SNARF	canine kidney (MDCK)	[34]
	7.64	YFP (SypHer)	HeLa	[38]
	7.6–8.2	GFP (mtAlpHi)	HeLa	[36]
	7.8	enhanced YFP	human bladder carcinoma	[45]
	7.7–8.2	pH-sensitive GFP	(Jurkat) T lymphocyte and HEK-293	[37]
	7.98	enhanced YFP	HeLa	[46]
	7.91	enhanced YFP	rat cardiomyocytes	[46]
	7.2–7.7	isotope distribution ^‡^	rat liver	[47]
	7.2–8.4	[U-^14^C]glutamate efflux	rat kidney	[48]
Na^+^	50–113 mM	CoroNa Red	canine kidney (MDCK)	[49]
	5 mM	SBFI	pig heart	[50]
	5.1 mM	SBFI	rat ventricular myocytes	[51]
	5 mM	assumed physiol. value	---------	[52]
K^+^	15 mM	PBFI	isolated rat hepatocytes	[53]
	10–20 mM	PBFI	rat heart	[54]
	20–60 mM ^†^	Potentiometric ISE	rat liver	[55]
	150 mM	assumed physiol. value	---------	[52]
Mg^2+^	0.35 mM	Mg^2+^ efflux null-point	rat hepatocytes	[56]
	0.9 mM	Mg^2+^ efflux null-point	beef heart	[57]
	2.4 mM	^31^P-NMR spectroscopy	heterotrophic sycamore	[58]
	0.5 mM	Fura-2	beef heart	[59]
	0.8–1.5 mM	Indo-1 and Fura-2	rat heart	[60]
Ca^2+^	0.22 µM	mito-TN-XXL	motor nerve of *Drosophila* fly larvae	[40]
	24 µM *	Rhod-FF/Rhod-5N	motor nerve of *Drosophila* fly larvae	[40]
	0.19–3.34 µM	YC2	HEK-293 and HeLa	[61]
	0.41 µM	aequorin luminescence	bovine adrenal glomerulosa cells	[62]
	183 nM	Indo-1	rat heart	[63]
	0.1–0.6 µM	Indo-1/AM	rat cardiac myocytes	[64]
	0.2–1.1 µM	Indo-1	rat heart	[65]
	0.17–0.92 µM	Fura-2/AM	rat heart	[66]
	0.2–1.8 µM	Fura-2	rat heart	[67]
	0.4–2.1 µM	Fura-2	rat heart myocytes	[68]
	1–5 µM	Fura-2/AM	rat brain	[29]
Cl-	4.2 mM	liquid chromatography	human liver	[69]
	0.9–22.2 mM	liquid chromatography	human liver	[69]

* axon firing; ^†^ results originally given in mmol/mg; ^‡^ distribution of [^14^C]− or [^3^H]acetate or [^14^C]5,5-dimethyloxazolidine-2,4-dione. Abbreviations: AM—acetoxymethyl ester, BCECF—2’,7’-biscarboxyethyl-5(6)-carboxyfluorescein, DMO—C-5-5-dimethyl-2,4-oxazolidinedione, GFP—green fluorescent protein, HEK-293—human embryonic kidney cells, Fura-2—calcium indicator C_29_H_22_N_3_O_14_K_5_, HeLa—human cervix-carcinoma cells, Indo-1—calcium indicator C_32_H_31_N_3_O_12_, MDCK—Madin-Darby Canine Kidney, PBFI—potassium-binding benzofuran isophthalate, SBFI—sodium-binding benzofuran isophthalate, SNARF—seminaphthorhodafluors fluorescence, YC2—Yellow Cameleon protein, YFP—yellow fluorescent protein.

(3) $$\ln\gamma_{i}=-\frac{Az_{i}{}^{2}\sqrt{I}}{1+a_{i}B\sqrt{I}}+\frac{(0.2-4.17\times 10^{-5}I)Az_{i}{}^{2}I}{\sqrt{1000}}$$

where$A=\frac{\sqrt{2}F^{2}e_{0}}{8\pi {\left(\varepsilon RT\right)}^{3/2}}$ and$B=\sqrt{\frac{2F^{2}}{\varepsilon RT}}$, *F* is the Faraday constant (96,488.46 C/mol), *R* is the ideal gas constant (8.3143 J/mol/K), T is the temperature (310 K), $e_{0}$ stands for the electronic charge (1.602 × 10^−19^ C), $\varepsilon =\varepsilon_{0}\varepsilon_{r}$ is the permittivity of the medium ($\varepsilon_{0}=8.854\times 10^{-12}$ F/m, $\varepsilon_{r}=80$ for water), and *a_i_* represents the radius of hydrated ion (values according to Kielland [70]).

**Figure 1:**
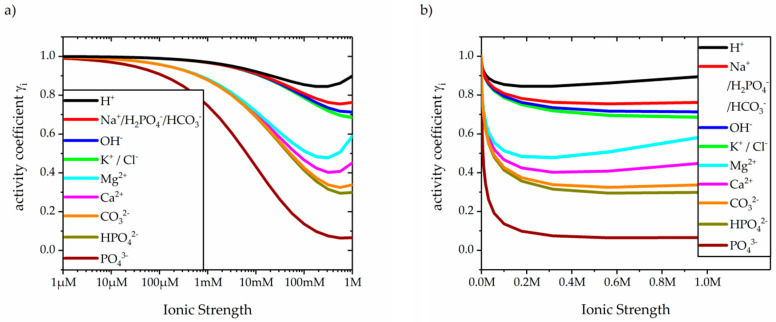
The calculated activity coefficients for the main inorganic ions present in mitochondria, (**a**) in logarithmic scale and (**b**) in linear scale.

As presented in Figure 1., in the ionic strengths *I* > 1 mM, the ideal solution assumption (*γ_i_* = 1) is not valid. In strong electrolytes, *I* > 200 mM, the activity coefficients (*γ_i_* ≠ 1) remain almost constant. In further calculations, the activity coefficients obtained for *I* = 0.7 M will be used.

### 2.2. Precipitation of Carbonates

Calcium and magnesium carbonates may form in the mitochondrial matrix due to the presence of carbon dioxide produced in citric acid cycle. Although the measurements of partial pressure of CO_2_ inside mitochondria is not yet possible with existing methods, it might be assumed to be equal or slightly higher than the partial pressure in blood. The partial pressure of carbon dioxide measured in human mixed venous blood is pCO_2_ = 6.1 kPa [71] to 6.3 kPa [72]. However, during exercises, it can rise to 7.8 kPa and after crossing the lactate acidosis threshold, it can reach values as high as 10.4 kPa [72]. 

The amount of gas dissolved in a solution is proportional to its partial pressure in the gas phase [73]. The proportionality factor is called the Henry’s law constant. Nevertheless, it should be kept in mind that its value still depends on certain parameters, e.g., temperature and the ionic strength of the solution. The Henry’s law constant for carbon dioxide dissolved in water at 25 °C equals K_H_ = 3.4 × 10^−4^ mol·m^−3^·Pa^−1^ [74]. It lowers with the increasing temperature reaching 2.5 × 10^−4^ mol·m^−3^·Pa^−1^ at 37 °C [75].

The hydration of carbon dioxide to form carbonic acid is slow to attain equilibrium below pH = 8 in pure systems. In biological systems the hydration of carbon dioxide is catalysed by carbonic anhydrase (a Zn-containing enzyme). Only a small portion of the aqueous carbon dioxide exists in its hydrated form as H_2_CO_3_. Hence, the relation between the carbonic species is usually expressed as:

(4) $$\left\{ \begin{array}{l} K_{1}\times {\{\mathrm{CO}}_{2}{(\mathrm{aq})\}=\{H}^{+}\}\times {\{\mathrm{HCO}}_{3}{}^{-}\}\\ K_{2}\times {\{\mathrm{HCO}}_{3}{}^{-}{\}=\{H}^{+}\}\times {\{\mathrm{CO}}_{3}{}^{2-}\} \end{array}\right.$$

where the equilibrium constants in pure water solutions at 25 °C equal K_1_ = 4.5 × 10^−7^, K_2_ = 4.7 × 10^−11^ [76]. 

These constants are temperature-dependent. Several different empirical equations have been developed to describe the relation between the equilibrium constants and temperature, including ones determined by Harned and co-workers [77,78,79], Ryzhenko [80], Millero and co-workers [81,82,83], as well as Plummer and Busenberg [75]. The values calculated using the latter at 37 °C, i.e., K_1_ = 4.96 × 10^−7^ and K_2_ = 5.73 × 10^−11^ are used in this work.

It should be noted, that mentioned equations are valid only for the solutions of carbon dioxide in pure water. The presence of other ions in the system lead to the increase of K_1_ and K_2_, e.g., values of equilibrium constants measured in sea water at 40 °C can reach K_1_ = 2.1 × 10^−6^ and K_2_ = 2.3 × 10^−10^ [83], comparing with calculated K_1_ = 5.1 × 10^−7^ and K_2_ = 6.0 × 10^−11^. In biological systems they can vary significantly and are treated not as true thermodynamic constants, but rather as apparent dissociation constants determined experimentally from the measurements of pH, pCO_2_ and dissolved CO_2_ [84]. Several studies [85,86,87,88,89] revealed the variability of K_1_ in human plasma in the range of 4.78 × 10^−7^ to 1.58 × 10^−6^. A case study of a diabetic child in ketoacidosis showed a change of K_1_ from 3.24 × 10^−6^ to 9.54 × 10^−7^ after just 7 h of treatment [90].

#### 2.2.1. Calcium Carbonates 

Ca^2+^ and CO_3_^2−^ ions form calcium carbonates CaCO_3_. There are six different forms of CaCO_3_, namely:

(1) calcite, anhydrous CaCO_3_ with trigonal structure
(2) aragonite, anhydrous CaCO_3_ with orthorhombic structure
(3) vaterite, anhydrous CaCO_3_ with hexagonal structure
(4) monohydrocalcite, CaCO_3_·H_2_O with trigonal structure
(5) hexahydrate known as ikaite, CaCO_3_·6H_2_O with monoclinic structure
(6) amorphous calcium carbonate (ACC), colloidal hydrate containing less than one molecule of water per molecule of CaCO_3_.

Although the first attempts to quantify the solubility of calcium carbonates in aqueous systems date back to the middle of the 19^th^ century [91,92] and despite the many efforts with current advanced ionic interaction models available, there are still no universally accepted values of the solubility constants of the calcium carbonate forms [93]. Values of the solubility products for various forms of CaCO_3_ and their activity products are presented in Table 2. Using these values, one can calculate the equilibrium concentration of free calcium in the solutions of respective salts as functions of pH and partial pressure of carbon dioxide.

#### 2.2.2. Magnesium Carbonates

Mg^2+^ and CO_3_^2−^ ions can form a variety of anhydrous, hydrated and basic magnesium carbonates, namely [99,100,101,102,103]:

(1) magnesite, anhydrous MgCO_3_ with trigonal crystal structure
(2) barringtonite, dihydrate MgCO_3_·2H_2_O with triclinic structure
(3) nesquehonite, trihydrate MgCO_3_·3H_2_O with monoclinic structure
(4) lansfordite, pentahydrate MgCO_3_·5H_2_O with monoclinic structure
(5) pokrovskite, basic carbonate Mg_2_CO_3_(OH)_2_ with monoclinic structure
(6) artinite, hydrated basic carbonate Mg_2_CO_3_(OH)_2_·3H_2_O with monoclinic structure
(7) hydromagnesite, hydrated basic carbonate Mg_5_(CO_3_)_4_(OH)_2_·4H_2_O with monoclinic structure
(8) dypingite, hydrated basic carbonate Mg_5_(CO_3_)_4_(OH)_2_·5H_2_O with monoclinic structure
(9) giorgiosite, hydrated basic carbonate Mg_5_(CO_3_)_4_(OH)_2_·6H_2_O
(10) octahydrate (UM1973-06-CO:MgH) with the formula Mg_5_(CO_3_)_4_(OH)_2_·8H_2_O, possibly identical to dypingite, but differs in optical properties
(11) protomagnesite, hydrated basic carbonate Mg_5_(CO_3_)_4_(OH)_2_·11H_2_O
(12) shelkovite, hydrated basic carbonate Mg_7_(CO_3_)_5_(OH)_4_·24H_2_O

Not all of the salts listed above can be precipitated directly from water solutions. Barringtonite is formed as the result of cold meteoric water percolating through olivine basalt and leaching magnesium from it [104]. Artinite is found along with hydromagnesite and other minerals as a low-temperature alteration product, as veinlets or crusts in serpentinized ultrabasic rocks [103]. Pokrovskite occurs within ultramafic bodies of dunite or serpentinite [105], and can be formed as an intermediate product in the thermal decomposition of artinite [106]. Shelkovite is a substance of anthropogenic origin, i.e., a product of burning coal mine dump [103]. 

Formation of the most stable anhydrous magnesite (MgCO_3_) is strongly kinetically inhibited because of the high hydration energy of Mg^2+^ [107,108]. The precipitation of magnesite is apparently inhibited at temperatures of less than 80 °C [109,110,111]. Therefore, various hydrous magnesium carbonates form instead. In water solutions, landsfordite, nesquehonite and hydromagnesite can precipitate, depending on the temperature. Lansfordite decomposes into nesquehonite at temperatures above 10 °C [112,113]. Most commonly, only the mineral nesquehonite can be precipitated from aqueous solutions at 25 °C and partial pressure of CO_2_ close to the ambient pressure or below [100,112,113,114,115,116]. At higher temperatures, i.e., above approximately 40 °C, various basic Mg-carbonates are usually formed by precipitation, mostly in the form of hydromagnesite [112,114,116,117].

The transformation from nesquehonite to hydromagnesite is unlikely to follow a single simple sequential reaction pathway [107] and a range of intermediate phases may form during this transformation [99]. Giorgiosite is a very poorly described basic magnesium carbonate [103,125] and it is considered as a metastable phase between nesquehonite and hydromagnesite [103]. Protohydromagnesite, a phase similar to dypingite, appears only as a metastable intermediate phase [114,126]. One intermediate phase is of particular interest, namely dypingite, a mineral that is known to be produced by cyanobacteria from alkaline wetlands [127]. Octahydrate Mg_5_(CO_3_)_4_(OH)_2_·8H_2_O is often considered a form of dypingite [120].

The reactions leading to the formation of the relevant magnesium carbonates together with the values of respective solubility products at 25 °C are summarized in Table 3. Several values obtained at 35 °C are also included in Table 3. Using these values, one can calculate the equilibrium concentration of free magnesium in the solutions of respective salts as functions of pH and partial pressure of carbon dioxide.

### 2.3. Precipitation of Orthophosphates 

Orthophosphate salts containing the PO_4_^3−^ group are distinguished from metaphosphates and pyrophosphates that contain PO_3_^−^ and P_2_O_7_^4−^ groups, respectively. The PO_4_^3−^ group takes part in ATP synthesis and occurs inside the mitochondrion in relatively high concentrations. Hence, the formation of metaphosphates and pyrophosphates will not be taken into consideration.

The concentration of phosphate ions is kept constant by the phosphate carrier while the relationship between them is given as:

(5) $$\left\{ \begin{array}{l} K_{1}\times {\{\;H}_{3}{\mathrm{PO}}_{4}{\}=\{H}^{+}\}\times {\{H}_{2}{\mathrm{PO}}_{4}{}^{-}\}\\ K_{2}\times {\{H}_{2}{\mathrm{PO}}_{4}{}^{-}{\}=\{H}^{+}\}\times {\{\mathrm{HPO}}_{4}{}^{2-}\}\\ K_{3}\times {\{\mathrm{HPO}}_{4}{}^{2-}{\}=\{H}^{+}\}\times {\{\mathrm{PO}}_{4}{}^{3-}\} \end{array}\right.$$

where K_1_ = 7.41 × 10^−3^, K_2_ = 6.31 × 10^−8^ and K_3_ = 4.07 × 10^−13^ [128].

#### 2.3.1. Calcium Orthophosphates

The calcium ortophosphates found in nature include [30,129,130,131]:

(1) monocalcium phosphate monohydrate (MCPM), Ca(H_2_PO_4_)_2_∙H_2_O with Ca/P ratio = 0.5
(2) anhydrous monocalcium phosphate (AMCP), Ca(H_2_PO_4_)_2_ with Ca/P ratio = 0.5
(3) dicalcium phosphate dihydrate, CaHPO_4_∙H_2_O_,_ known as brushite (BRU), Ca/P ratio = 1.0
(4) anhydrous dicalcium phosphate, CaHPO_4,_ known as monetite, Ca/P ratio = 1.0
(5) octacalcium phosphate (OCP) with the formula Ca_8_(HPO_4_)_2_(PO_4_)_4_∙5H_2_O, Ca/P ratio = 1.33
(6) α-tricalcium phosphate (α-TCP), Ca_3_(PO_4_)_2_ with monoclinic crystallographic structure and Ca/P ratio = 1.5
(7) β-tricalcium phosphate (β-TCP), Ca_3_(PO_4_)_2_ with rhombohedral crystallographic structure and Ca/P ratio = 1.5
(8) amorphous calcium phosphates (ACP), Ca_x_H_y_(PO_4_)_z_·nH_2_O, n = 3 to 4.5, Ca/P ratio = 1.2 to 2.2
(9) calcium-deficient hydroxyapatite (CDHA), Ca_10−x_(HPO_4_)_x_(PO_4_)_6−x_(OH)_2−x_ (0 < x ≤ 2) with Ca/P ratio = 1.5 to 1.67
(10) hydroxyapatite (HA) with the formula Ca_10_(PO_4_)_6_(OH)_2_, Ca/P ratio = 1.67
(11) chloroapatite (ClA) with the formula Ca_10_(PO_4_)_6_(Cl)_2_, Ca/P ratio = 1.67
(12) carbonated apatite (CO_3_A), Ca_10_(PO_4_)_6_CO_3,_ known as dahllite, Ca/P ratio = 1.67
(13) fluoroapatite (FA), with the formula Ca_10_(PO_4_)_6_F_2_ and Ca/P ratio = 1.67
(14) oxyapatite (OA), with the formula Ca_10_(PO_4_)_6_O and Ca/P ratio = 1.67
(15) tetracalcium phosphate (TetCP), Ca_4_(PO_4_)_2_O, known as hilgenstockite, Ca/P ratio = 2.0

Another calcium salt, i.e., isoclasite with the formula Ca_2_(PO_4_)OH·2H_2_O, has been reported in 1870 by Sandberger [132] as orthorhombic crystals. His measurement was rather inaccurate (the angle between prismatic faces measured by contact goniometer) and the crystals could be hexagonal, possibly of apatite. Doubts concerning validity of data on isoclasite are supported by the absence of any additional description of isoclasite anywhere [133]. 

Some of the calcium salts mentioned above, including tetracalcium phosphate, oxyapatite, α-tricalcium phosphate and β-tricalcium phosphate, cannot be precipitated from aqueous solutions [131]. Anhydrous monocalcium phosphate and anhydrous dicalcium phosphate are stable only in temperatures above 100 °C, while monocalcium phosphate monohydrate is stable only at pH < 2 [131]. Energy dispersive x-ray spectra from mitochondrial precipitates do not show the presence of fluor [27]. Hence, the formation of fluoroapatite can be ruled out as well.

The formulas of the calcium phosphates that might precipitate in the mitochondrion matrix, together with the values of respective solubility products at 37 °C are summarized in Table 4 (some of the literature values have been recalculated to correspond to the activity product given in the table). To calculate the equilibrium concentration of chloroapatite and carbonated apatite, the concentrations of chlorides and carbonates have to be provided. The free chloride concentration in the mitochondrial matrix equals [Cl^−^] = 4.2 mM [69]. The concentration of carbonate ions [CO_3_^2−^] are calculated using Equation (4), assuming that partial pressure of carbon dioxide equals pCO_2_ = 7 kPa. 

#### 2.3.2. Magnesium Orthophosphates

The magnesium ortophosphates found in nature include [142]:

(1) anhydrous trimagnesium phosphate, Mg_3_(PO_4_)_2_ with monoclinic crystal structure, occurs as the natural mineral farringtonite
(2) trimagnesium phosphate octahydrate, Mg_3_(PO_4_)_2_·8H_2_O with monoclinic structure, occurs as the natural mineral babierrite
(3) trimagnesium phosphate docosahydrate, Mg_3_(PO_4_)_2_·22H_2_O with triclinic structure, occurs as the natural mineral cattiite
(4) anhydrous dimagnesium phosphate, Mg(HPO_4_)
(5) dimagnesium phosphate monohydrate, Mg(HPO_4_)·H_2_O
(6) dimagnesium phosphate trihydrate, Mg(HPO_4_)·3H_2_O with orthorhombic structure, occurs as the natural mineral newberyite
(7) dimagnesium phosphate heptahydrate, Mg(HPO_4_)·7H_2_O with monoclinic structure, occurs as the natural mineral phosphorrösslerite
(8) anhydrous monomagnesium phosphate, Mg(H_2_PO_4_)_2_
(9) monomagnesium phosphate dihydrate, Mg(H_2_PO_4_)_2_·2H_2_O
(10) monomagnesium phosphate tetrahydrate, Mg(H_2_PO_4_)_2_ ·4H_2_O
(11) magnesium hydroxyphosphate, Mg_2_(PO_4_)(OH) with trigonal structure, occurs as the natural mineral holtedahlite
(12) magnesium ammonium phosphate, NH_4_MgPO_4_·6H_2_O with orthorhombic structure, occurs as the natural mineral struvite
(13) magnesium potassium phosphate, KMgPO_4_·6H_2_O

Due to the high hydration energy, the direct precipitation of anhydrous magnesium phosphates is not possible. The anhydrous Mg(HPO_4_) compound is formed by dehydration at 100–170 °C [142]. The anhydrous farringtonite is formed by heating its hydrates at 400 °C [142].

Monomagnesium phosphate and its hydrates dissolve in water, forming phosphoric acid and depositing a solid precipitate of Mg(HPO_4_)·3H_2_O [142], e.g., precipitation monomagnesium phosphate tetrahydrate requires a non-aqueous solvent in which H_3_PO_4_ is soluble but Mg(H_2_PO_4_)_2_·4H_2_O has limited solubility [143]. Among dimagnesium phosphates only the trihydrate is stable in the MgO/H_2_O/P_2_O_5_ system at room temperature [142]. The trimagnesium phosphate docosahydrate is metastable and reverts to octahydrate in water [144].

It has been shown that increased levels of ammonia disturb mitochondrial function, induce oxidative stress, causes mitochondrial membrane potential collapse and negatively affects several key enzymes responsible for energy metabolism [145,146,147,148]. Although the mitochondria are responsible for the production of NH_4_^+^ ions during the oxidation of glutamate [149], in healthy mitochondria the concentration of ammonia is very low. Hence, the formation of struvite can be ruled out. Furthermore, in the biological precipitation of struvite, Ca^2+^ ions make struvite precipitation difficult and even inhibit it [150,151].

The reactions leading to the formation of the relevant magnesium phosphates together with the values of respective solubility products at 38 °C are summarized in Table 5. Using these values, the equilibrium concentration of phosphates [∑P_i_]_eq_, required for the formation of each magnesium orthophosphate can be calculated.

### 2.4. Precipitation of Polyphosphates 

It has been reported that PO_4_^3−^ can be concentrated and stored by biochemical polymerization into polyphosphate (polyP) ions [157]. They are negatively charged polyanions (P_n_O_3n+1_)^(n+2)−^ with great affinity for calcium and other multivalent cations [158]. PolyP is a macroergic compound so that the energy of phosphoanhydride bond hydrolysis is the same as in ATP [159]. Polyphosphates have been found in all organisms ranging from bacteria to mammals [160]. 

The synthesis of polyP is mainly realized by the polyphosphate kinase, which catalyses the reaction ATP + [PO_3_^−^]_n_ ↔ ADP + [PO_3_^−^]_n+1_. PolyP can play a role in the energetic metabolism of mitochondria [161], e.g., it has been shown that, in human cell lines, specific reduction of mitochondrial polyP under expression of yeast exopolyphosphatase PPX1 significantly modulates mitochondrial bioenergetics and transport [159]. Polyphosphates (including pyrophosphate) have also a significant role in the regulation of the intensity of several biosynthetic processes in mitochondrial and cell energy metabolism [162,163].

PolyP can form several insoluble salts. Bobtelsky and Kertes [164], have analysed the properties of pyrophosphates (P_2_O_7_)^4−^ and tripolyphosphates (P_3_O_10_)^5−^ and the precipitation of calcium, magnesium, barium and strontium salts of these ions. With the addition of calcium to the respective polyP solution, the insoluble Ca_2_P_2_O_7_ and Ca_5_(P_3_O_10_)_2_ will form, while the addition of magnesium leads to the precipitation of Na_2_Mg_3_(P_2_O_7_)_2_ and NaMg_2_P_3_O_10_, respectively. Interestingly, the redissolution of these salts will take place if the excess of the polyphosphate ions is added to the solution [164].

Polyphosphate chelation of calcium reduces the free calcium concentration [165] and produces a neutral, amorphous, [Ca(PO_3_)_2_]_n_ complex. Discrete granules containing calcium–polyP complexes have been observed e.g., in acidocalcisomes, the electron-dense acidic organelles that have been conserved from bacteria to humans [166]. PolyP are noted to be highly inhibitory to calcium phosphate nucleation and precipitation [167], e.g., they inhibit crystalline calcium hydroxyapatite nucleation and growth from solution [168]. However, in basic pH solutions calcium polyphosphate glass releases hydrated calcium and polyphosphate ions into solution and, through hydrolytic degradation of polyP, calcium polyphosphate eventually transforms into hydroxyapatite [169]. The relationships between polyphosphate chemistry and apatite biomineralization as well as the possible role of polyP in mitochondria have been discussed in detail by Omelon et al. [165,170].

The important polyP forms in living cells are specific complexes with polyhydroxybutyrate (PHB) and Ca^2+^, which have been found in the membranes of many organisms, including membranes of mitochondria of animal cells [171,172]. The PHB forms a helix with a lipophilic shell of methyl groups and polar lining of ester carbonyl oxygens surrounding a core helix of polyP with Ca^2+^ bridging the two polymers. These helices can form voltage-dependent, Ca^2+^-selective channels, or be components of ion-conducting proteins: namely, the human erythrocyte Ca^2+^-ATPase pump and the *Streptomyces lividans* potassium channel [173].

The polyP pool in mitochondria likely consists of two parts, i.e., one part localized directly in the membranes as a polyP–PHB–Ca^2+^ complex, and the other part present in the intermembrane space and not bound to PHB, though its association with cations, including Ca^2+^, is not improbable [159]. It was determined by subfractionation of isolated mitochondria by mild osmotic treatment, that intermembrane space contained ~90% of total mitochondrial polyP [174]. This suggests that polyphosphates are formed outside the mitochondrial matrix. Furthermore, the formation of calcium polyP inside the matrix seems less likely than calcium orthophosphates, because the Ca/P ratio of this complex is less than 1, while the mitochondrial Ca/P ratio is between 1 and 2.

## 3. Results

### 3.1. Precipitation of Calcium Carbonates

**Figure 2:**
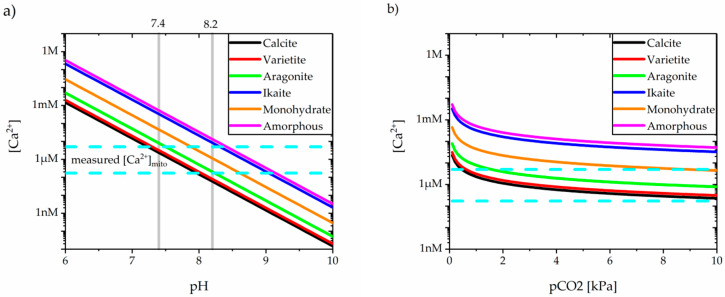
The calculated equilibrium concentrations of free calcium (**a**) as functions of pH at 37 °C and pCO_2_ = 7 kPa, (**b**) as a function of partial pressure of carbon dioxide at 37 °C and pH = 7.8.

Using the values presented in Table 2, one can calculate the equilibrium concentration of free calcium in the solutions of respective salts as functions of pH and partial pressure of carbon dioxide. Such calculations are shown in Figure 2.

The more stable crystalline forms are generally those with a lower solubility product, hence lower equilibrium concentration of calcium. However, the formation and transformation of solid phases during the spontaneous precipitation of calcium carbonate from alkaline, carbonate-rich solutions is a highly complicated and time-dependent process. The local conditions of temperature, concentration and impurities can have a profound effect on the qualitative nature of the phases formed, as well as on the quantitative kinetics of individual processes.

The Ostwald-Lussac Law of Stages states that, in the pathway to the final crystalline state, will pass through all less stable states in order of increasing stability [175]. The formation of metastable phases is a quite common phenomenon during spontaneous precipitation of supersaturated solutions [98,176,177]. During the precipitation of more stable anhydrous forms, amorphous calcium carbonate, calcium carbonate hexahydrate and calcium monohydrocalcite appear as intermediate phases of importance and consequence. At high supersaturation, the first-formed phase is ACC, a rather unstable precursor consisting of spherical particles of calcium carbonate [94,98]. ACC changes to more stable forms via dissolution and precipitation. The solubility product of ACC is higher than for other forms of CaCO_3_. Hence, any solution in equilibrium with the amorphous phase will be highly supersaturated with respect to the crystalline phases, which leads to nucleation-controlled growth [178]. In other words, ACC serves as a precursor for other polymorphs, the nucleation of the polymorphous crystals occurs in the vicinity of the clouded precursor and the crystals grow by consuming the precursors surrounding the crystals [179].

If magnesium ions are present in the reaction system, they become incorporated into the ACC structure. The crystalline phase, which forms from the amorphous precursor, is controlled by the magnesium content of the precursor. Pure crystallises to vaterite [180], ACC containing 10% Mg crystallises to calcite [181], ACC with 30% crystallises to monohydrocalcite [178,182] and ACC with 50% Mg crystallises to protodolomite/dolomite (at temperatures higher than 60 °C) [183]. 

High concentrations of orthophosphates prevent the crystallization of the more stable anhydrous forms of CaCO_3_, which leads to the occurrence of ikaite and monohydrocalcite in the system [94,98]. Ikaite, however, for its formation requires near-freezing conditions and is unstable in the temperatures above 25 °C [184]. Soluble acidic proteins occurring in the mitochondrial matrix might also have a strong inhibiting effect on the nucleation of anhydrous polymorphs, e.g., it has been shown that poly-l-aspartic acid completely inhibits the formation of varietite [185]. Na^+^ and Cl^−^ ions exert a weak or no influence on the precipitation of carbonates [186,187].

The presence of both magnesium and orthophosphate ions in mitochondrial matrix suggests that monohydrocalcite might be the phase occurring inside mitochondria. However, the solubility calculations presented in Figure 2, clearly show that monohydrocalcite can form only if matrix pH is greater than 7.8 (for [Ca^2+^] = 5 µM) or even 8.6 (for [Ca^2+^] = 0.17 µM). In lower pH, neither monohydrocalcite, nor any other calcium carbonate will precipitate.

### 3.2. Precipitation of Magnesium Carbonates

**Figure 3:**
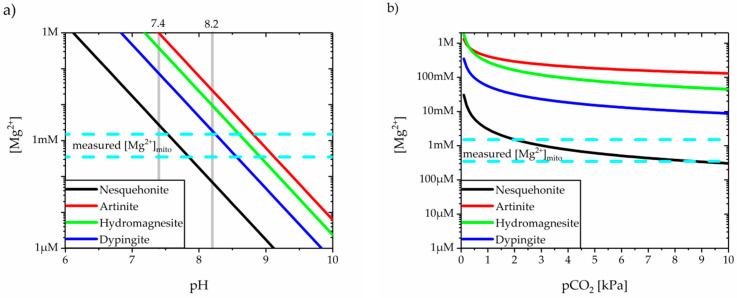
The calculated equilibrium concentrations of free magnesium (**a**) as functions of pH at 35 °C and pCO_2_ = 7 kPa, (**b**) as a function of partial pressure of carbon dioxide at 35 °C and pH = 7.8.

Using the solubility products presented in Table 3, one can calculate the equilibrium concentration of free magnesium in the solutions of respective salts as functions of pH and partial pressure of carbon dioxide. Solubility calculations, presented in Figure 3, clearly show that nesquehonite is the most stable magnesium carbonate in given conditions. It is also the only magnesium carbonate that can precipitate in the mitochondrial matrix, but only if the matrix pH is greater than 7.5 (for [Mg^2+^] = 1.5 mM) or even 7.9 (for [Mg^2+^] = 0.35 mM). In lower pH, neither nesquehonite, nor any other magnesium carbonate will precipitate.

### 3.3. Precipitation of Calcium Orthophospates

**Figure 4:**
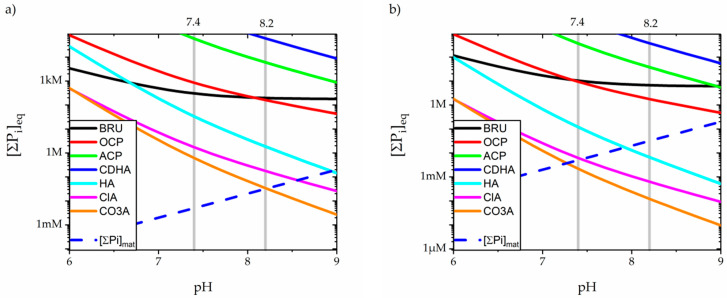
The calculated equilibrium concentrations [∑P_i_]_eq_ required for the formation of calcium phosphates as functions of pH. Calcium concentration set to (**a**) [Ca^2+^] = 0.17 µM and (**b**) [Ca^2+^] = 5 µM.

The total concentration of phosphates [∑P_i_]_eq_, required for the formation of each salt at constant free calcium concentration [Ca^2+^] = 0.17 µM and [Ca^2+^] = 5 µM, is presented in Figure 4. Salts with the lowest [∑P_i_]_eq_ are thermodynamically most stable and therefore dominate in the system, i.e., whenever the total concentration of free phosphates exceeds the lowest [∑P_i_]_eq_, formation of the respective salt will occur, keeping [∑P_i_] at a constant level. As shown in Figure 4, carbonated apatite is the most stable calcium salt, if [Ca^2+^] is kept in the micromolar range.

Assuming the constant concentration of phosphates in the cytoplasm [∑P_i_]_cyt_ = 2 mM, and that the equilibrium at phosphate translocase yields [∑P_i_]_mat_/[∑P_i_]_cyt_ = [H^+^]_cyt_/[H^+^]_mat_, one can calculate the total concentration of mitochondrial phosphates [30]. The calculated values of [∑P_i_]_mat_ compared with the [∑P_i_]_eq_ of the respective salts (see Figure 4b) clearly show that if the calcium concentration is elevated, the solution of the mitochondrion matrix (pH = 7.4 to 8.2) is supersaturated with respect to up to three salts. i.e., hydroxyapatite, chloroapatite and carbonated apatite. Other salts (OCP, brushite, CDHA and ACP) having [∑P_i_]_eq_ > [∑P_i_]_mat_ will dissolve, hence cannot form inside mitochondria.

Calcium apatites (hydroxyapatite, chloroapatite, fluoroapatite and carbonated apatite) are members of a large family of more than 75 chemically different apatites, i.e., compounds with the chemical formula: A_2_B_3_(XO_4_)_3_Y, where A and B are bivalent cations, and XO_4_ and Y are trivalent and monovalent anions, respectively [188]. The high-symmetry members of the apatite family crystallise in the hexagonal system.

Calcium apatites are very often non-stoichiometric, due to the substitution of OH^−^ in hydroxyapatite with Cl^−^, F^−^ and CO_3_^2−^. Therefore, the formulas for carbonated apatite and chloroapatite are often given as Ca_10_(PO_4_)_6_(CO_3_)_x_(OH)_2−2x_ and Ca_10_(PO_4_)_6_Cl_x_(OH)_2__−__x_, respectively [139,140]. The apatite structure allows varied substitutions of calcium and phosphate ions to take place without a significant alteration in its basic structure, e.g., one of the basic phosphorite minerals, known as francolite, has a variable chemical composition, which can be represented by (Ca,Mg,Sr,Na)_10_(PO_4_,SO_4_,CO_3_)_6_F_2+x_, where 0 < x < 1 [189,190].

Formation of CO_3_^2−^-substituted hydroxyapatite is common in biological and geological systems and this substitution has been well documented in several studies [191,192,193]. Carbonate ion in the structure of biological apatites can substitute either the OH^−^ ion (type A carbonated apatite) or PO_4_^3−^ ion (type B carbonated apatite) [194] and these two locations of carbonate species can be distinguished using spectroscopic methods [195,196]. The carbonate group distorts the apatite lattice and causes the resulting solid to be considerably more soluble than hydroxyapatite [197]. The substitution of carbonate for phosphate would also reduce the crystallinity and precipitation rate of HA [198]. Another specificity of biological apatites is the presence of hydrogen phosphate (HPO_4_^2−^) ions in PO_4_^3−^ sites [199,200,201]. These two types of bivalent ions substituting for PO_4_^3−^ have been shown to correspond to the formation of calcium deficient apatites, i.e., the loss of a negative charge due to these substitutions is compensated by the creation of cationic vacancies and anionic vacancies in the OH^−^ site [202]. The formula for biological apatites taking into account the possible existence of both type A and B substitutions reads [203,204]:

Ca_10−x_(PO_4_)_6−x_(HPO_4_ or CO_3_)_x_(OH or ½CO_3_)_2−x_ with 0 < x < 2. (6)

This general formula can be used, e.g., to represent the bone composition at all ages in many vertebrates (x = 1.7) or a typical composition of human tooth enamel (x = 0.6) [203]. The Ca/P ratio of carbonated apatite vary from 2.0 (x = 2 and all type B substitutions with CO_3_^2−^) down to 1.2 (x = 2 and all type B substitutions with HPO_4_^2−^) or even lower if cationic substitutions (2Na^+^ or Mg^2+^ for Ca^2+^) occur. 

Precipitated apatite crystals generally exhibit a structured hydrated surface layer containing mainly bivalent cations and anions. This feature should not be confused with the Stern double layer that forms on most mineral surfaces. The substitution possibilities in the hydrated layer are not well known but seem to be greater than in the core of the apatite structure [203]. Magnesium ions for example, which hardly penetrate the apatite lattice, are easily and reversibly incorporated into the hydrated layer in large amounts [205]. Ion substitutions in the hydrated layer considerably modify its structure even though these alterations seem reversible in most cases [206].

### 3.4. Precipitation of Magnesium Orthophospates

**Figure 5:**
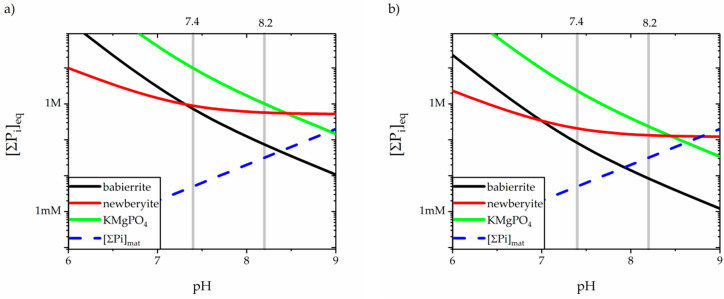
The calculated equilibrium concentrations [∑P_i_]_eq_ required for the formation of magnesium phosphates as functions of pH. Magnesium concentration set to (**a**) [Mg^2+^] = 0.35 mM and (**b**) [Mg^2+^] = 1.5 mM.

The solubility calculations for magnesium orthophosphates at low magnesium concentration [Mg^2+^] = 0.35 mM (see Figure 5a) clearly show that solution of mitochondrial matrix is undersatured with the respect to all magnesium phosphate salts. Hence, they cannot precipitate. At high magnesium concentrations [Mg^2+^] = 1.5 mM, the precipitation of babierrite is possible if pH > 7.9 (see Figure 5b). In lower pH, neither babierrite, nor any other magnesium phosphate will precipitate.

## 4. Discussion

The results of solubility calculations (presented in Figure 2, Figure 3, Figure 4 and Figure 5) show that four different salts can form in the mitochondrion, depending on the changes in ionic concentrations induced by the metabolic processes in mitochondria and by the concentration changes in cytosol. Babierrite can precipitate at high magnesium concentration and pH > 9. Nesquehonite precipitates at pH > 7.5 (or pH > 7.8 if calcium concentration is low). Monohydrocalcite precipitates at high Ca^2+^ concentrations and pH > 7.8. Carbonated apatite at high calcium concentrations precipitates in a whole mitochondrial pH range, and dissolves when mitochondrial [Ca^2+^] is low. However, these calculations do not take into account the influence of magnesium ions on precipitation of calcium salts and vice versa. They also neglect the influence of phosphate ions on the precipitation of carbonate salts and the influence of carbonate ions on the precipitation of phosphate salts. These relations are discussed below.

### 4.1. Carbonates in the Ca/Mg System

Insoluble salts containing both calcium and magnesium are known as dolomites, i.e., the family of non-stoichiometric compounds with the formula Ca_1−x_Mg_1+x_(CO_3_)_2_ [207,208,209,210]. Sedimentary dolomites with x < 0 are named calcium dolomites, and the ones with x > 0 are named magnesian dolomites. The carbonate dominated by calcite is called limestone and the mineral Ca_0.5_Mg_1.5_(CO_3_)_2_ (x = 0.5) is named huntite. Other carbonates: ankerite Ca(Fe, Mg, Mn)(CO_3_)_2_ and kutnohorite Ca(Mn,Mg,Fe)(CO_3_)_2_, with bivalent cations: Fe^2+^ and Mn^2+^, substituting a part of Mg^2+^ ions in the layered structure, also enter the dolomite group [211].

The hydration shell of magnesium is more strongly bound than the hydration shell of calcium [108,212]. Hence, the energy required to desolvate magnesium is higher than that of calcium. That explains the high temperatures required to crystallize anhydrous Mg-Ca carbonates, i.e., temperature of 60 °C is required for the formation of dolomite [183] and repeated trials to precipitate it under laboratory conditions at room temperature were unsuccessful [213,214]. Another anhydrous carbonate with two cations, namely eitelite with the formula Na_2_Mg(CO_3_)_2_, can also be precipitated only at temperatures above 60 °C [215].

The hydrated nature of monohydrocalcite means that full dehydration of Mg^2+^ is not required before incorporation into the crystal lattice. Therefore, it will more likely form than the anhydrous calcium carbonate phases [178]. Monohydrocalcite has a high capacity to accommodate magnesium within its structure. The coordination number of Ca^2+^ in monohydrocalcite is 8 [216,217] and Mg^2+^ does not take a coordination number other than 6. Therefore, Mg^2+^ is not incorporated into the monohydrocalcite structure, directly but forms discrete hydrated magnesium carbonate insertions. According to the solubility calculations presented in Figure 2 and Figure 3, the solution in mitochondrial matrix is saturated with respect to both monohydrocalcite and nesquehonite, if pH > 8. It is in agreement with the observations of Nishiyama et al. [182], who have shown that in Ca^2+^/Mg^2+^/CO_3_^2−^ systems nesquehonite with very low crystallinity is expected to be formed with monohydrocalcite and the amorphous material. The hydrous magnesium carbonates surrounding monohydrocalcite play a protective role in preventing its dehydration to anhydrous calcium carbonates.

High-Mg monohydrocalcite consists of individual nanometer-sized crystals (<35 nm) with a significant part of its structural H_2_O being associated with magnesium [178]. X-ray diffraction (XRD) experiments show that monohydrocalcite exhibits low crystallinity and small particle size when the ratio of magnesium to calcium in the solid is higher than 0.4, i.e., the increase of magnesium content lead to broad X-ray diffraction peaks with low intensity. Monohydrocalcite with Mg/Ca ratio higher than 0.5 exhibits the properties characteristic for amorphous material (no XRD peaks are observed) [182]. This corresponds well with the results of the electron microscopy measurements of brain and liver mitochondria that show a lack of crystal structures in the matrix [27]. The value of solubility of amorphous Mg-rich monohydrocalcite is slightly higher than in the case of pure monohydrocalcite [182]. Hence, in order to precipitate, it requires the solution to be more alkaline than the mitochondrial matrix.

### 4.2. Carbonated Apatite in Physiological Solutions

The studies on slow spontaneous precipitation of calcium carbonates and calcium phosphates under physiological conditions [218], show that calcium carbonate precipitation is prevented by the presence of phosphate ions at concentrations too low for the precipitation of calcium phosphate. Such precipitation is prevented, when the phosphate/bicarbonate concentration ratio is higher than about l/300. Above this ratio, the competition of the phosphate ions with the carbonate ions is sufficient to inhibit the nucleation and crystal growth of calcium carbonates, while the phosphate concentration is still too low for the nucleation of phosphates [218]. Pyrophosphate and hexametaphosphate ions inhibit the precipitation of calcium carbonate in a similar way. Hence, the composition of most biological fluids does not allow the deposition of calcium carbonate and it is suggested that such deposition can occur only under the influence of metabolic processes which locally raise the bicarbonate and lower the phosphate concentration [218].

Carbonate ions may affect the solubility of phosphate salts in several ways [191]. Ion pair complexes between calcium and bicarbonate and carbonate will form and the presence of ion pairs and reduction of free Ca^2+^ would increase the soluble concentration of phosphate. However, the calculations indicate that ion pairs will be the predominant calcium-containing species at pH greater than 8.5 and at high carbonate levels (0.05 M) [219]. The carbonate ions may also compete with phosphate for sites on the growing crystallites. Nucleation and crystal growth may be hindered by the extra time and energy required to eliminate carbonate from phosphate sites and vice versa. When crystal growth does take place, carbonate can readily substitute for phosphate in the apatite lattice, as discussed earlier. Chemical analyses reveal that the apatitic tissues always contain smaller or larger amounts of carbonate which does not crystallize separately as carbonate [197].

Precipitation of babierrite is also inhibited, because of the presence of carbonate and calcium ions. Due to the chemical similarity between Mg^2+^ and Ca^2+^, magnesium co-precipitates with calcium phosphates instead, inducing the formation of an amorphous phase rather than a stable crystalline carbonated apatite [191,220]. Magnesium ions stabilize the amorphous precipitates of calcium carbonate phosphate. Apatitic precipitates are more poorly-crystallized when formed in the presence of magnesium ions than when formed in their absence, and the crystallinity of the precipitates formed in the presence of magnesium decreases with rising [Mg^2+^]. The Ca/P ratios of the amorphous precipitates are often significantly lower when formed in the presence of Mg^2+^ ions, and the equilibrium concentrations of calcium and phosphates are higher when the precipitates are amorphous, than when they are crystalline [221].

Biological calcium apatites show a broad range of chemical compositions but also structure. It has been shown that hydroxyapatite precipitates obtained in the conditions where pH is kept constant show low crystallinity [222]. In primitive organisms, calcium apatites are generally amorphous, while in more elaborated organisms, especially in vertebrates, calcium apatites can crystallise [203]. Magnesium has been shown to disturb the crystallization of calcium apatites from solution when used at concentrations sufficient to be a major competitor for calcium [223]. The growth of apatite crystals is impeded by the Mg^2+^ ions, because they block the active growth sites and interrupt the crystal structure [224]. 

Organic compounds present in the mitochondrial matrix have a similar effect on phosphate crystals formation. Namely, the nucleotide diphosphates and triphosphates, including ADP and ATP, as well as low molecular weight metabolites containing two attached ester phosphate groups such as citrate also have the ability to inhibit the crystallization of calcium phosphates and may function as in vivo mineralization inhibitors [225,226]. Adsorption of organic compounds on the newly-formed nuclei results in a small crystal size and could be a factor in producing less crystalline phase [198]. Although apatite is usually considered a crystallographic structure, we use the term amorphous carbonated apatite to distinguish it from the amorphous calcium phosphate with the formula Ca_x_H_y_(PO_4_)_z_.

A characteristic property of apatite compounds is the ability to incorporate ions and individual molecules during its synthesis. One of the most interesting examples is the molecular oxygen, which forms inside the structure through the decomposition of unstable precursor: peroxide ions and hydrogen peroxide molecules trapped in the apatite structure [227]. Mitochondria are involved in the cell’s response to oxidative stress, hence the ability of apatite to entrap H_2_O_2_ and convert it to O_2_ might have had a crucial role in the evolution of mitochondria, especially in the early stages before the ability to create enzyme glutathione peroxidase has been developed.

Apatite can incorporate a large variety of foreign ions, including sodium, potassium, strontium, barium, manganese, zinc, iron, cobalt, nickel and even rare earth elements (see [228] for a detailed review). This property is used by a few organisms to eliminate toxic elements and survive in polluted environments, e.g., in gastropods *Littorina Littorea*, apatites allow the accumulation and biochemical deactivation of excess quantities of cadmium and other potentially harmful cations [229,230]. Small organic molecules, such as formate ions and glycine molecules, can also be incorporated in apatites [202,203]. It has been shown that amorphous granular aggregates forming in mitochondria include glycoproteins in their structure [231].

## 5. Summary and Conclusions

The solubility calculations for calcium and magnesium salts, including carbonates and phosphates, led to the identification of four salts that may form in the mitochondrial matrix: monohydrocalcite, nesquehonite, carbonated apatite and babierrite. Further analysis of inhibition properties of several inorganic and organic ions present in the mitochondrial matrix led to a conclusion, that non-stoichiometric magnesium-rich carbonated apatite precipitates in the matrix under physiological conditions. 

The formation of crystalline apatites is inhibited by the presence of magnesium ions, ATP, ADP and citrates. Therefore, amorphous and low-crystalline carbonated apatite is precipitated. Low crystallinity of the precipitated material can be attributed to numerous ionic substitutions in the apatite structure. The general chemical formula of this salt can be expressed as:

(Ca,Mg)_10−x_(PO_4_)_6−x_(HPO_4_,CO_3_)_x_(OH,½CO_3_,Cl)_2−x_ with 0 < x < 2. (7)

Numerous ions and molecules can also be incorporated in the structure of apatite, which leads to a further decrease of the crystallinity of precipitated material. The ability to incorporate hydrogen peroxide and decompose it to molecular oxygen might have an auxiliary role in the response to oxidative stress.

Precipitation and dissolution of carbonated apatite inside the matrix, affects the mitochondrial ionic concentrations, and therefore indirectly influences the action of several protein entities in the inner mitochondrial membrane including e.g., ATP synthase, phosphate translocase, calcium uniporter (MCU), mitoK_ATP_ and mitoK_Ca_ channels as well as the K^+^/H^+^, Na^+^/H^+^ and Ca^2+^/3Na^+^ exchangers.

When the oxidative phosphorylation chain is active, the membrane potential on the inner mitochondrial membrane is highly negative, which causes the influx of calcium to the matrix through MCU and leads to the precipitation of carbonated apatite. The dominant forms of carbonic and phosphoric acid in the mitochondrial pH range are HCO_3_^−^ and HPO_4_^2−^, respectively. Hence, precipitation of carbonated apatite leads to the production of protons, e.g.,:

10Ca^2+^ + HCO_3_^−^ + 6HPO_4_^2−^ → Ca_10_(PO_4_)_6_CO_3_ + 7H^+^ (10 − x)Ca^2+^ + xHCO_3_^−^ + (6 − x)HPO_4_^2−^ + (2 − x)H_2_O ↔ Ca_10−x_(PO_4_)_6−x_(CO_3_)_x_(OH)_2−x_ + (8 − x)H^+^ (8)

These protons are then pumped outside the mitochondrial matrix by the oxidative phosphorylation chain, which allows the production of ATP.

In anaerobic conditions, i.e., during hypoxia, the oxidative phosphorylation chain stops. Hypoxia can be caused e.g., by ischemia (low blood flow) or carbonate monoxide poisoning (oxygen in hemoglobin replaced by CO). There are three phases of ATP production during ischemia [232]. At first, up to 16 min ATP is produced due to glycolysis cytoplasm acidifies but there is no increase in sodium (5 mM) or calcium concentration (140 nM) in the cytoplasm, i.e., Na-K-ATPase of the cytoplasmic membrane is fully active. In the second phase, as hypothesised by Dołowy [30], during hypoxia, the proton motive force is replaced by the calcium-motive force caused by the dissolution of calcium phosphates, efflux of calcium ions and alkalization of the matrix due to the following processes:

Ca_10_(PO_4_)_6_CO_3_ + 7H_2_O → 10Ca^2+^↑ + CO_2_↑ + 6HPO_4_^2−^ + 8OH^−^ Ca_10−x_(PO_4_)_6−x_(CO_3_)_x_(OH)_2−x_ + 6H_2_O → (10 − x)Ca^2+^↑ + xCO_2_↑ + (6 − x)HPO_4_^2−^ + 8OH^−^ (9)

where ↑ represents ions or molecules leaving the mitochondrial matrix. Phosphate ions forming due to the dissolution of carbonated apatite and lower ATP/ADP ratio are the two factors responsible for lowering the free energy of the ATP synthesis, hence allowing its production in hypoxic conditions. During the second phase which lasts up to 30 min, sodium concentration in the cytoplasm is slowly rising to 30 mM and calcium concentration to 500 nM level. The mitochondrion can survive, as long as it can keep the pH of the matrix constant by the precipitation and dissolution of carbonated apatite. Then during the third phase, when all available salt dissolves, further influx of protons leads to the acidification of the matrix, hence lowering the proton motive force below the level necessary for ATP production. When matrix acidifies Na/H and K/H exchangers cannot prevent influx of osmotically active cations into the matrix and mitochondria swells. Low ATP levels in the cytoplasm lead to opening of the mitoK_ATP_ channel. Upon reperfusion sharp increase in mitochondrial electric potential leads to further influx of osmotically active cations into the mitochondrial matrix and formation of the permeability transition pore [233,234].

The build-up of amorphous carbonated apatite should increase the maximum time of hypoxia that the cell can survive. It explains the increased life-time observed in the heart cells preconditioned by short increases of the calcium concentration [235,236]. Calcium preconditioning increases the cytoplasmic calcium ion concentration. Therefore Ca^2+^ from the cytoplasm is more rapidly transported into the matrix, which leads to the precipitation of carbonated apatite. A better understanding of the relationship between calcium storage and the mechanisms of ischemic preconditioning may lead to the development of new stroke-protective drugs.

## Figures and Tables

**Table 2:** The precipitation of insoluble calcium carbonates. The activity products (IAP) and the solubility products K_sp_ (at 37 °C) are obtained from the literature.

	Formula	Activity Product (*Q*)	Solubility Product, K_sp_ at 37 °C	Ref.
Calcite	CaCO_3_	{Ca^2+^} {CO_3_^2−^}	3.31 × 10^−9^ *	[75]
			3.27 × 10^−9^ to 4.02 × 10^−9^	[93]
Aragonite	CaCO_3_	{Ca^2+^} {CO_3_^2−^}	4.57 × 10^−9^ *	[75]
			4.67 × 10^−9^ to 5.41 × 10^−9^	[93]
Varietite	CaCO_3_	{Ca^2+^} {CO_3_^2−^}	1.23 × 10^−8^ *	[75]
			1.18 × 10^−8^ to 1.84 × 10^−8^	[93]
Ikaite	CaCO_3_·6H_2_O	{Ca^2+^} {CO_3_^2−^}	2.40 × 10^−7^ *	[94]
			3.50 × 10^−7^	[95]
Monohydro-calcite	CaCO_3_·H_2_O	{Ca^2+^} {CO_3_^2−^}	7.09 × 10^−8^ *	[96]
			2.51 × 10^−8^	[97]
ACC	CaCO_3_·nH_2_O	{Ca^2+^} {CO_3_^2−^}	9.09 × 10^−7^ *	[94]
	(n < 1)		3.98 × 10^−7^	[98]

* Values used in the calculations presented in this work.

**Table 3:** The precipitation of insoluble magnesium carbonates. The activity products (*Q*) and the solubility products K_sp_ (at 25 °C) are obtained from the literature.

	Formula	Activity Product (*Q*)	Solubility Product, K_sp_ at 25 °C	Ref.
Nesquehonite	MgCO_3_·3H_2_O	{Mg^2+^} {CO_3_^2−^}	2.38 × 10^−6^	[99]
			1.10 × 10^−5^	[118]
			2.57 × 10^−6^	[119]
			5.37 × 10^-6^	[120]
			4.57 × 10^−6^ *^†^	[120]
			5.90 × 10^−5^	[121]
Artinite	Mg_2_CO_3_(OH)_2_·3H_2_O	{Mg^2+^}^2^ {CO_3_^2−^} {OH^−^}^2^	6.31 × 10^−18^	[119]
			7.76 × 10^−18^ *	[122]
Hydromagnesite	Mg_5_(CO_3_)_4_(OH)_2_·4H_2_O	{Mg^2+^}^5^ {CO_3_^2−^}^4^ {OH^−^}^2^	6.31 × 10^−31^	[119]
			1.25 × 10^−32^	[123]
			3.16 × 10^−33^ *^†^	[123]
			8.32 × 10^−38^	[124]
Dypingite	Mg_5_(CO_3_)_4_(OH)_2_·nH_2_O	{Mg^2+^}^5^ {CO_3_^2−^}^4^ {OH^−^}^2^	1.12 × 10^−35^	[120]
	(n = 5 or 8)		9.12 × 10^−37^ *^†^	[120]

* Values used in the calculations presented in this work; ^†^ K_sp_ obtained at 35 °C.

**Table 4:** The precipitation of insoluble calcium orthophosphates. The activity products (*Q*) and the solubility products K_sp_ (at 37 °C) are obtained from the literature.

	Formula	Activity Product (*Q*)	Solubility Product, K_sp_ at 37 °C	Ref.
BRU	CaHPO_4_	{Ca^2+^} {HPO_4_^2−^}	2.34 × 10^−7^ *	[129]
			2.26 × 10^−7^	[134]
			0.92 × 10^−7^	[135]
OCP	Ca_8_(HPO_4_)_2_(PO_4_)_4_	{Ca^2+^}^8^ {H^+^}^2^ {PO_4_^3−^}^6^	1.25 × 10^−96^ *	[129]
			1.20 × 10^−97^	[134]
			3.98 × 10^−98^	[136]
			2.51 × 10^−99^	[137]
ACP	Ca_x_H_y_(PO_4_)_z_	{Ca^2+^}^x^ {H^+^}^y^ {PO_4_^3−^}^z^	2.0 × 10^−26^ *^†^	[130]
		{Ca^2+^}^3^ {PO_4_^3−^}^2^	2.0 × 10^−33^–1.6 × 10^−25^	[29]
		{Ca^2+^} {H^+^}^0.22^ {PO_4_^3−^}^0.74^	2.3 × 10^−11^, 3.2 × 10^−12 §^	[138]
CDHA	Ca_10-x_(HPO_4_)_x_(PO_4_)_6-x_(OH)_2-x_	{Ca^2+^}^10−x^ {H^+^}^x^ {PO_4_^3−^}^6^ {OH^−^}^2−x^	~7.94 × 10^−86^ *	[130]
HA	Ca_10_(PO_4_)_6_(OH)_2_	{Ca^2+^}^10^ {PO_4_^3−^}^6^ {OH^−^}^2^	1.00 × 10^−118^	[29]
			6.31 × 10^−118^ *	[129]
			2.00 × 10^−119 ‡^	[139]
			2.51 × 10^−111^	[140]
			5.42 × 10^−119^	[141]
ClA	Ca_10_(PO_4_)_6_Cl_2_	{Ca^2+^}^10^ {PO_4_^3−^}^6^ {Cl^−^}^2^	3.98 × 10^−116^ *	[140]
CO_3_A	Ca_10_(PO_4_)_6_CO_3_	{Ca^2+^}^10^ {PO_4_^3−^}^6^ {CO_3_^2−^}	1.58 × 10^−103^ *^‡^	[139]

* Values used in the calculations presented in this work; ^†^ K_sp_ is pH-dependent, value in the table for pH = 7.4; ^‡^ K_sp_ obtained at 25 °C; ^§^ authors observed two different metastable phases.

**Table 5:** The precipitation of insoluble magnesium phosphates. The activity products (*Q*) and the solubility products K_sp_ (at 38 °C) are obtained from the literature.

	Formula	Activity Product (*Q*)	Solubility Product, K_sp_ at 38 °C	Ref.
Babierrite	Mg_3_(PO_4_)_2_·8H_2_O	{Mg^2+^}^3^ {PO_4_^3−^}^2^	6.31 × 10^−28^	[152]
			5.37 × 10^−26^ *^†‡^	[144]
			2.00 × 10^−26 §^	[153]
Newberyite	Mg(HPO_4_)·3H_2_O	{Mg^2+^} {HPO_4_^2−^}	3.47 × 10^−5^	[154]
			1.78 × 10^−6^ *^†^	[144]
			2.40 × 10^−6 ‡^	[144]
			1.78 × 10^−6 §^	[155]
			1.48 × 10^−6 §^	[153]
Magnesium potassium phosphate	KMgPO_4_·6H_2_O	{K^+^} {Mg^2+^} {PO_4_^3−^}	2.4 × 10^−11 §^	[156]

* Values used in the calculations presented in this work; ^†^ recalculated based on [152]; ^‡^ recalculated based on [154]; ^§^ K_sp_ obtained at 25 °C.
